# Angiopoietin-like 4 promotes osteosarcoma cell proliferation and migration and stimulates osteoclastogenesis

**DOI:** 10.1186/s12885-018-4468-5

**Published:** 2018-05-08

**Authors:** T. Zhang, A. Kastrenopoulou, Q. Larrouture, N. A. Athanasou, H. J. Knowles

**Affiliations:** 1 Botnar Research Centre, Nuffield Department of Orthopaedics Rheumatology and Musculoskeletal Sciences, University of Oxford, Headington, Oxford, OX3 7LD UK; 2 State Key Laboratory of Oral Disease, West China Hospital of Stomatology, Sichuan University, Chengdu, 610041 People’s Republic of China

**Keywords:** Angiopoietin-like 4 (ANGPTL4), Osteosarcoma, Proliferation, Migration, Colony formation, Osteoclastogenesis, Bone resoprtion

## Abstract

**Background:**

Osteosarcoma is the most common primary bone cancer in children and young adults. It is highly aggressive and patients that present with metastasis have a poor prognosis. Angiopoietin-like 4 (ANGPTL4) drives the progression and metastasis of many solid tumours, but has not been described in osteosarcoma tissue. ANGPTL4 also enhances osteoclast activity, which is required for osteosarcoma growth in bone. We therefore investigated the expression and function of ANGPTL4 in human osteosarcoma tissue and cell lines.

**Methods:**

Expression of ANGPTL4 in osteosarcoma tissue microarrays was determined by immunohistochemistry. Hypoxic secretion of ANGPTL4 was tested by ELISA and Western blot. Regulation of ANGPTL4 by hypoxia-inducible factor (HIF) was investigated using isoform specific HIF siRNA (HIF-1α, HIF-2α). Effects of ANGPTL4 on cell proliferation, migration (scratch wound assay), colony formation and osteoblastogenesis were assessed using exogenous ANGPTL4 or cells stably transfected with ANGPTL4. Osteoclastogenic differentiation of CD14+ monocytes was assessed by staining for tartrate-resistant acid phosphatase (TRAP), bone resorption was assessed by lacunar resorption of dentine.

**Results:**

ANGPTL4 was immunohistochemically detectable in 76/109 cases. ANGPTL4 was induced by hypoxia in 6 osteosarcoma cell lines, under the control of the HIF-1α transcription factor. MG-63 cells transfected with an ANGPTL4 over-expression plasmid exhibited increased proliferation and migration capacity and promoted osteoclastogenesis and osteoclast-mediated bone resorption. Individually the full-length form of ANGPTL4 could increase MG-63 cell proliferation, whereas N-terminal ANGPTL4 mediated the other pro-tumourigenic phenotypes.

**Conclusions:**

This study describes a role(s) for ANGPTL4 in osteosarcoma and identifies ANGPTL4 as a treatment target that could potentially reduce tumour progression, inhibit angiogenesis, reduce bone destruction and prevent metastatic events.

**Electronic supplementary material:**

The online version of this article (10.1186/s12885-018-4468-5) contains supplementary material, which is available to authorized users.

## Background

Osteosarcoma is the most common primary bone tumour in children and young adults. It is a highly aggressive tumour which rapidly invades and destroys the surrounding bone and metastasises through the vasculature early in the course of disease. Although survival has improved in the past two decades for patients with non-metastatic osteosarcoma, those with either metastatic disease at diagnosis or with recurrent disease have a poor prognosis, with a 5 year survival rate of only 30% [1]. There is therefore an urgent need for new targeted therapies to improve survival of patients with metastatic or recurrent disease.

Micro-environmental hypoxia and expression of the HIF transcription factor are common features of osteosarcoma which strongly correlate with disease progression, recurrence and poor survival [2–8]. Both isoforms of HIF (HIF-1α, HIF-2α) promote osteosarcoma cell proliferation and migration in vitro [9–11]. In vivo studies with microRNAs or HIF inhibiting drugs have shown that HIF inhibition reduces tumour growth [12, 13] and overcomes chemo-resistance of osteosarcoma [14]; conversely HIF-1α over-expression enhances tumourigenicity [15]. However current HIF inhibitors are not specific to the HIF pathway.

ANGPTL4 is a secreted adipokine, expression of which is regulated by hypoxia and HIF-1α [16–18]. It was originally described as a central regulator of lipid metabolism and the primary physiological regulator of lipoprotein lipase activity [19, 20], under the control of the PPAR family of transcription factors [21–23]. It is a blood-borne hormone directly involved in regulating glucose homeostasis, lipid metabolism and insulin sensitivity [24, 25].

There are three isoforms of ANGPTL4. Full-length ANGPTL4 (flANGPTL4) is the largest isoform, which can be proteolytically cleaved at an internal linker region by proprotein convertases to generate N-terminal coiled-coil domain (nANGPTL4) and C-terminal fibrinogen-like domain (cANGPTL4) fragments [26]. This cleavage process is tissue dependent; the human liver secretes cleaved ANGPTL4, whereas adipocytes secrete the full-length form [21, 27]. The three forms of ANGPTL4 exert distinct physiological functions, regulation of lipid metabolism being the primary function of nANGPTL4 (20, 22).

ANGPTL4 expression is elevated in many epithelial tumours [28–30] where it is associated with metastasis and reduced survival [31–35]. Most experimental literature also describes ANGPTL4 as pro-tumourigenic. ANGPTL4 stimulates tumour cell proliferation [36], supports anchorage-independent growth and confers anoikis resistance [29, 37], so promoting in vivo tumourigenesis [29, 36]. ANGPTL4 is also pro-angiogenic and is thought to facilitate metastasis by enhancing endothelial permeability [18, 28, 30, 38, 39]. ANGPTL4 expression has not been described in osteosarcoma tissue, although as a HIF target gene it is likely that ANGPTL4 is over-expressed in osteosarcoma and may correlate with features of tumourigenesis or metastasis.

ANGPTL4 also stimulates osteoclast activity to increase the rate of bone resorption [16, 40]. Osteoclasts are multi-nucleated cells specialised to perform lacunar resorption of bone, over-activation of which results in osteolytic disease. Osteoclasts are often observed within the primary tumour in osteosarcoma [41, 42], within which increased expression of osteoclast activity genes positively associates with tumour aggressiveness and metastatic disease [42, 43]. Tumour-induced osteolysis, mediated by osteoclasts, is a characteristic feature of osteosarcoma and use of osteoclast-targeted agents is a therapeutic strategy for treatment of the disease [44, 45]. Increased osteoclast activity might also mediate the reduced bone mineral density and osteoporosis often detected in osteosarcoma patients even after successful treatment [46].

Investigating the expression and function of ANGPTL4 in osteosarcoma will provide fundamental information as to its suitability as a target for the treatment of osteosarcoma. This is particularly relevant as such a treatment would potentially target the primary tumour, inhibit angiogenesis, reduce metastatic events and prevent bone destruction.

## Methods

### Materials

Tissue culture reagents were from Lonza (Cheltenham, UK) except FBS (Invitrogen, Paisley, UK). MCSF, full-length ANGPTL4, c-terminal and n-terminal ANGPTL4 were from R&D Systems (Abingdon, UK); RANKL was produced in-house. Unless stated, other reagents were from Sigma-Aldrich (Poole, UK).

### Immunohistochemistry

Osteosarcoma tissue microarrays were prepared in-house. All samples were collected with donor-informed consent. This project was approved by the Oxford Clinical Research Ethics Committee (C01.070). Antigen retrieval of de-waxed and rehydrated paraffin-embedded samples was performed in boiling 1 mM EDTA (pH 8). Slides were incubated in anti-ANGPTL4 (H200; Santa Cruz Biotechnology, Heidelberg, Germany) or a PBS control for 1 h. Immunostaining was performed using the VECTASTAIN Elite Universal ABC Kit and DAB (Vector Laboratories). Digital images of slides were obtained using an Olympus BX40 microscope. Database searching of the R2: Genomics Analysis and Visualization Platform (http://r2.amc.nl) was performed using the programme available on the website.

### Osteosarcoma cell culture

Osteosarcoma cell lines MG63, MHM, HOS-143B, IOR-OS18, HOS, ZK-58 and OSA were obtained from the EuroBoNeT cell line biobank (http://eurobonet.pathobiology.eu/cd/index.php), comprising fully characterised bone tumour cell lines [47], and maintained in RPMI 1640 medium supplemented with 10% FBS, L-glutamine (2 mM), penicillin (50 IU/ml) and streptomycin sulphate (50 μg/ml). No ethical approval was required for their use. For establishment of ANGPTL4 stable transfectants, osteosarcoma cell lines were transfected with an ANGPTL4 ORF expression clone (W0165-M02; GeneCopoeia) or the corresponding empty vector control (EV). Following clonal selection, transfected cell lines were maintained in RPMI 1640 additionally supplemented with G418 (0.1 mg/ml).

### HIF small interfering RNA (siRNA)

To silence expression of HIF-1α or HIF-2α, siRNA sequences against the corresponding genes or a HIF-1α scrambled control [48] were transfected into osteosarcoma cell lines at 30 nM concentration using RNAiMAX (Invitrogen). Culture medium was removed after 24 h and cells were incubated in normoxia or hypoxia (0.5% O_2_) for another 18 h.

### Protein analysis

Whole cell proteins were extracted in lysis buffer (6.2 M urea, 10% glycerol, 5 mM DTT, 1% SDS, protease inhibitors), separated by SDS-PAGE and transferred onto a PVDF membrane. Primary antibodies were against HIF-1α (clone 54, BD Biosciences, Oxford, UK), HIF-2α (EP190b; Abcam, Cambridge, UK), ANGPTL4 (H200; Santa Cruz Biotechnology, Heidelberg, Germany) and β-tubulin. Secondary antibody was goat anti-mouse or -rabbit IgG-HRP. Proteins were visualized by chemiluminescence. ANGPTL4 secretion was measured using the human ANGPTL4 DuoSet (R&D Systems) according to the manufacturer’s instructions. ANGPTL4 secretion was normalized to cell number as quantified by crystal violet staining.

### Proliferation assay

Cells were seeded at 1 × 10^3^ cells/well in 96-well plates in media containing 0.5% FBS. If required, exogenous ANGPTL4 was added every 3–4 days. Crystal violet staining was used to determine cell number at day 1, 3, 5 and 7. Cells were fixed with 4% formalin, incubated with 1% crystal violet for 60 min at 37 °C, then lysed in 0.2% Triton X-100 before the absorbance was read at 550 nm.

### Scratch assay

Cells were seeded in 12-well plates. When confluence reached 80%, a sterilized pipette was used to scratch the cell monolayer. After washing away cell debris, cells were maintained in media containing 1% FBS. Specific points of the scratch were photographed at 0 and 24 h. Wound width was measured in ImageJ and migration expressed as fraction wound closure.

### Colony formation

Cells were seeded in 6-well plates at the density of 150 cells per well and maintained for 14 days. If required, exogenous ANGPTL4 was added every 3–4 days. Cells were then fixed in 4% formalin, stained with crystal violet and air dried.

### Osteoblastic differentiation of osteosarcoma cell lines

Cells were seeded in 24-well plates until 80% confluent and then treated with 100 nM dexamethasone, 5 mM β-glycerophosphate and 50 μg/ml ascorbic acid for 3 weeks, changing the medium every 3–4 days. If required, exogenous ANGPTL4 was added at each media change. Cells were fixed for alkaline phosphatase staining using naphthol AS-MX phosphate as a substrate and reaction of the product with fast violet B salt. For quantification staining was solubilised with isopropanol and quantified at 400 nm. Mineralization was visualized by Alizarin red staining. Quantification was achieved by extraction in 10% acetic acid, neutralization in 10% NH_4_OH and measurement at 405 nm.

### Monocyte proliferation and osteoclast differentiation

Primary human monocytes were obtained from peripheral blood mononuclear cells isolated from leucocyte cones (NHS Blood and Transplant, UK). CD14 positive cells were selected using CD14 MicroBeads for magnetic cell sorting (Miltenyi Biotech). For proliferation assays, monocytes were incubated for 9 days in α-MEM medium (without ribonucleosides / deoxyribonucleosides, containing 10% FBS, 2 mM L-glutamine, 50 IU/ml penicillin and 50 μg/ml streptomycin sulphate) containing either exogenous ANGPTL4 or 10% MG-63 conditioned media. Cell number was assessed by crystal violet staining. For osteoclast differentiation, CD14-selective monocytes were maintained in αMEM supplemented with 25 ng/ml MCSF and 15 ng/ml RANKL as well as either exogenous ANGPTL4 or 10% MG-63 conditioned media for 10 days. A 35 ng/ml RANKL treatment was used as a positive control. Formalin-fixed cells were stained for tartrate-resistant acid phosphatase (TRAP) using naphthol AS-BI phosphate as a substrate with reaction of the product with Fast Violet B salt. Multi-nucleated cells containing three or more nuclei were considered osteoclasts. Resorption of dentine discs was visualized by staining with 0.5% toluidine blue, following the removal of adherent cells by sonication. Dentine discs were photographed, resorption tracks highlighted, and the resorbed area was quantified using ImageJ.

### Statistics

All results are presented as mean ± SD of at least three independent experiments. One-way ANOVA using Bonferroni’s multiple comparison as a post hoc test or t-test for only two comparisons was used for statistical analysis.

## Results

### ANGPTL4 is expressed in osteosarcoma tissue and is hypoxia-inducible in osteosarcoma cell lines

**Fig. 1:**
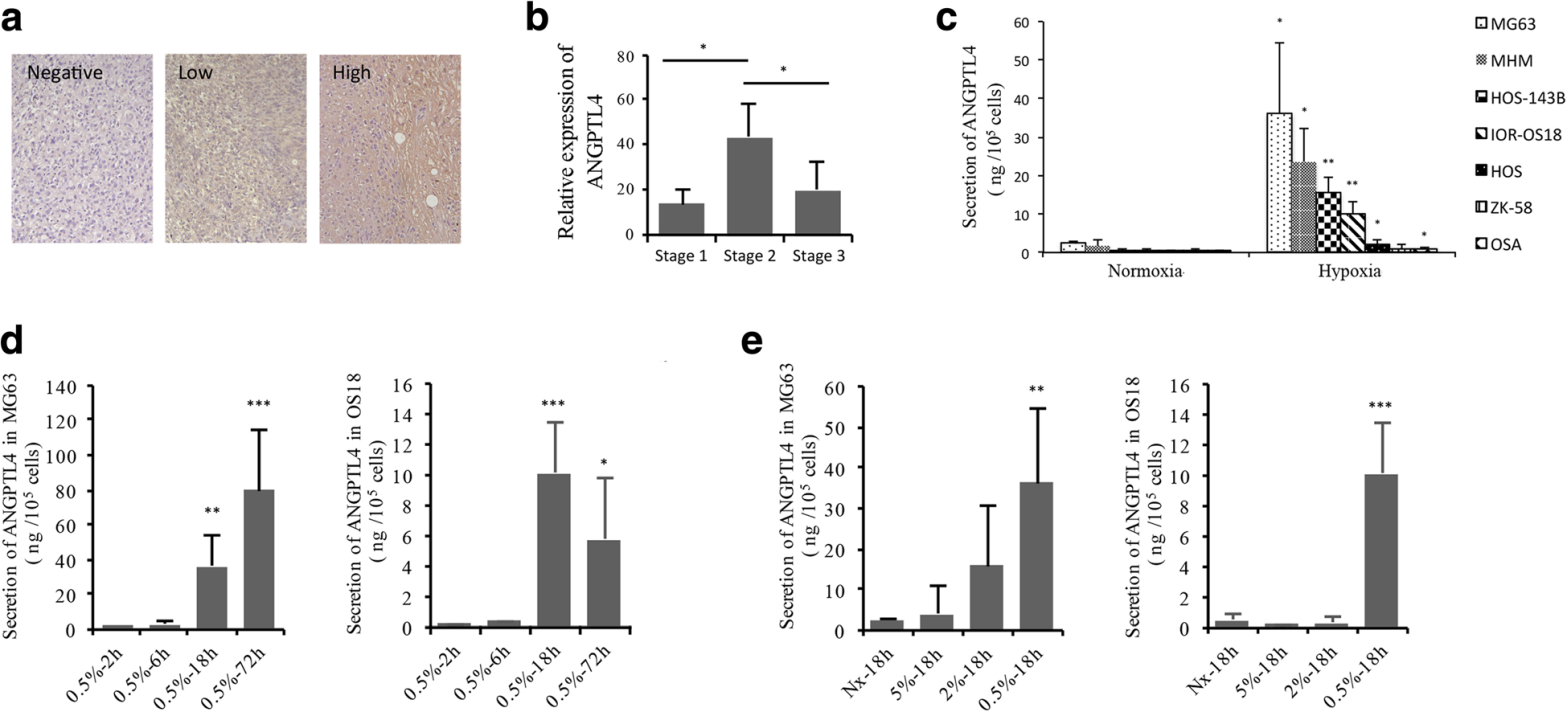
ANGPTL4 is expressed in osteosarcoma and is hypoxia-inducible in osteosarcoma cell lines **a** 76/109 osteosarcomas expressed ANGPTL4, including 51 with low level expression and 25 with high level expression. **b** ANGPTL4 mRNA is high in stage 2 osteosarcoma (pre-metastatic osteosarcoma that has spread beyond the bone). **c** Hypoxia (0.5% O_2_, 18 h) induced ANGPTL4 secretion in six osteosarcoma cell lines (MG-63, MHM, HOS-143B, IOR-OS18, HOS and OSA). **d**, **e** ANGPTL4 secretion by MG-63 and OS18 cells increased in a (**d**) time- and (**e**) oxygen concentration-dependent manner. *p < 0.05, **p < 0.005, ***p < 0.001 versus the corresponding normoxic control

To determine whether ANGPTL4 is expressed in osteosarcoma, ANGPTL4 immunohistochemistry was performed on an osteosarcoma tissue microarray (TMA). 76/109 osteosarcomas expressed ANGPTL4, with 51 tumours showing low level expression and 25 high level expression (Fig. 1a). Clinical and patient data is not available for the tissues in this TMA; it was therefore not possible to tell whether ANGPTL4 expression levels correlated with clinical characteristics of osteosarcoma. However, searching publicly available databases indicated increased expression of ANGPTL4 mRNA in grade 2 osteosarcomas versus either low grade tumours or those that had metastasised (Fig. 1b).

Seven different osteosarcoma cell lines (MG-63, MHM, HOS-143B, IOR-OS18, HOS, ZK-58 and OSA) were assessed for basal secretion of ANGPTL4 which, with the exception of MG-63 and MHM cells, was almost undetectable. As ANGPTL4 is a hypoxia-induced adipokine, we next exposed the cell lines to different duration (2 h, 6 h, 18 h and 72 h) and intensity (5, 2 and 0.5% O_2_) of hypoxia. MG-63 and MHM cells showed the highest hypoxic secretion of ANGPTL4 (Fig. 1c). ANGPTL4 secretion increased in a time- and oxygen concentration-dependent manner (Fig. 1d, e).

### Hypoxic induction of ANGPTL4 is regulated by HIF-1α and HIF-2α in osteosarcoma cells

**Fig. 2:**
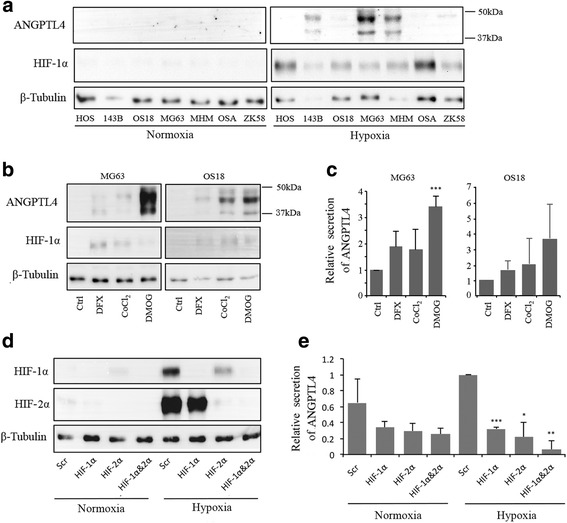
ANGPTL4 is induced by HIF-1α and HIF-2α in osteosarcoma cells (**a**) Expression of HIF-1α and full-length ANGPTL4 was assessed in 7 osteosarcoma cell lines exposed to hypoxic conditions (0.5% O_2_, 18 h). **b**, **c** Induction of ANGPTL4 in MG-63 and OS18 cells was observed after 18 h exposure to dimethyl oxalyl glycine (DMOG, 1 mM) by both (**b**) western blot and (**c**) ELISA. **d** Western blot showing complete knockdown of HIF-1α and HIF-2α protein in hypoxic MG-63 cells using isoform-specific HIF-1α and HIF-2α siRNA. **e** Effect of HIF-1α and HIF-2α siRNA on ANGPTL4 secretion in normoxic and hypoxic MG-63 . *p < 0.05; **p < 0.005; ***p < 0.001 versus the corresponding normoxic or hypoxic control

Induction of hypoxia was confirmed by stabilisation of HIF-1α protein in all 7 osteosarcoma cell lines. Intracellular ANGPTL4 was only detectable in those cell lines with the greatest hypoxic secretion of ANGPTL4 (Fig. 2a, Fig. 1c). HIF pathway activation with dimethyl oxalyl glycine (DMOG) also increased ANGPTL4 expression, as observed by western blot (Fig. 2b) and ELISA (Fig. 2c). Hypoxic induction of ANGPTL4 is regulated by HIF-1α in most cell types [16–18]. Isoform-specific HIF-1α and HIF-2α siRNA achieved complete knockdown of HIF -1α and HIF-2α protein in hypoxic MG-63 cells (Fig. 2d). Exposure to either HIF-1α siRNA, HIF-2α siRNA or HIF-1α plus HIF-2α siRNA suppressed secretion of ANGPTL4 under hypoxia, showing that ANGPTL4 is a target gene of both HIF-1α and HIF-2α in osteosarcoma cells (Fig. 2e).

### ANGPTL4 enhances osteosarcoma cell proliferation and migration

**Fig. 3:**
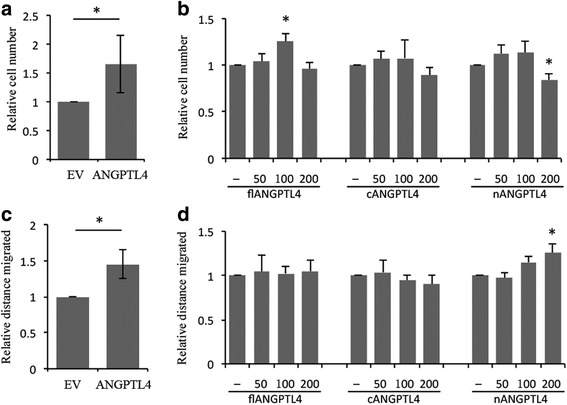
ANGPTL4 enhances osteosarcoma cell proliferation and migration (**a**) ANGPTL4 over-expressing MG63 cells exhibited a higher proliferation rate, which was reproduced by exposure to (**b**) 100 ng/ml exogenous flANGPTL4. (**c**) ANGPTL4 over-expressing MG63 cells had an increased migration capacity, which was reproduced by exposure to (**d**) 200 ng/ml exogenous nANGPTL4. *p < 0.05 compared to corresponding control groups

We next considered whether ANGPTL4 might enhance the tumourigenic properties of osteosarcoma cells, as seen in other tumour types. MG-63 cells stably transfected with an ANGPTL4 expression plasmid (MG63-A4) showed 10-fold over-expression of ANGPTL4 (51.6 ng/10^5^ cells) compared to control cells (MG63-EV; 5.0 ng/10^5^ cells) (Additional file 1: Figure S1). MG63-A4 cells showed an increase in proliferation rate (Fig. 3a) and migration capacity (Fig. 3c), although with no effect on plating efficiency in colony formation assays. We therefore investigated whether flANGPTL4, nANGPTL4 or cANGPTL4 was the primary mediator of these effects. No individual isoform had as strong an effect on the tumourigenic properties of MG-63 cells as seen in the MG63-A4 transfectants. However flANGPTL4 induced MG-63 cell proliferation (Fig. 3b) and nANGPTL4 induced MG-63 cell migration (Fig. 3d). ANGPTL4 knock-down in shRNA transfected cells had no effect on any parameter tested, presumably due to the low levels of expression of ANGPTL4 under standard cell culture conditions (Additional file 1: Figure S1, Fig. 1c).

### ANGPTL4 affects osteoblastic differentiation of osteosarcoma cells

**Fig. 4:**
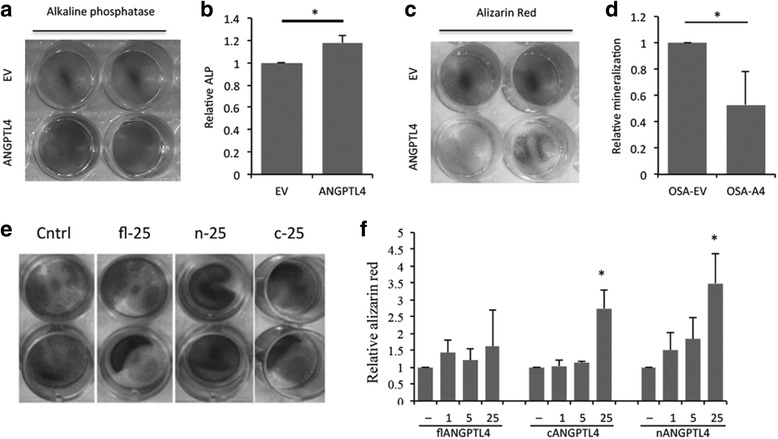
ANGPTL4 affects osteoblastic differentiation of osteosarcoma cells (**a**, **b**) ANGPTL4 over-expressing OSA cells showed increased ALP production but (**c**, **d**) reduced mineralization as revealed by Alizarin Red staining. (**e**, **f**) Low concentrations (25 ng/ml) of the cleaved forms of ANGPTL4 (cANGPTL4 and nANGPTL4) promoted mineralization of OSA cells. *p < 0.05

A common characteristic of osteosarcoma cells is the ability to form mineralised osteoid and differentiate down the osteoblastic lineage [49]. On exposure of the panel of osteosarcoma cell lines to osteoblastogenic culture medium, only three cell lines (HOS, OSA and ZK58) were capable of osteoblastic differentiation. We therefore made OSA stable transfectants over-expressing ANGPTL4 (OSA-A4), which showed 90-fold over-expression of ANGPTL4 (126.8 ng/10^5^ cells) compared to control cells (OSA-EV; 1.4 ng/10^5^ cells). Production of alkaline phosphatase and mineralization, representing early and late stages of osteoblast differentiation, was measured at day 14 and day 21 respectively. OSA-A4 cells showed increased early osteoblastic differentiation (Fig. 4a, b) but a reduced ability to mineralise (Fig. 4c, d). No individual isoform of ANGPTL4 affected ALP production (data not shown) but a low concentration (25 ng/ml) of the cleaved forms of ANGPTL4 (cANGPTL4 and nANGPTL4) promoted mineralization of OSA cells (Fig. 4e, f).

### ANGPTL4 promotes monocyte proliferation and enhances osteoclast differentiation and bone resorption

**Fig. 5:**
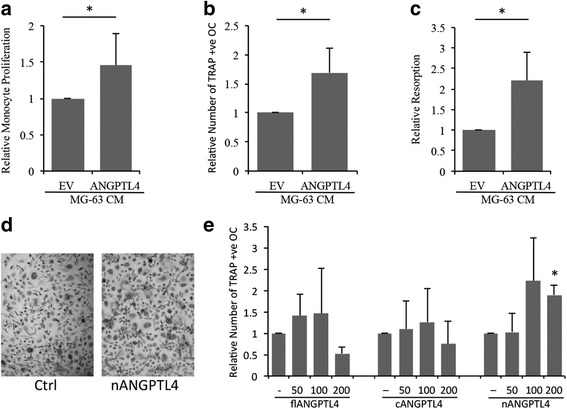
ANGPTL4 promotes monocyte proliferation and enhances osteoclast differentiation (**a**) Proliferation of monocytes and (**b**) formation of TRAP positive osteoclasts was increased by 10% conditioned media collected from ANGPTL4 over-expressing cells. (**c**) MG63-A4 conditioned medium caused elevated bone resorption of mature osteoclasts. (**d**, **e**) Quantification of the number of TRAP-positive multi-nucleated osteoclasts formed following treatment with different concentrations (50, 100, 200 ng/ml) of flANGPTL4, cANGPTL4 and nANGPTL4. *p < 0.05

Osteosarcoma cell lines secrete osteoclastogenic factors that are able to stimulate the formation of multi-nucleated osteoclasts from monocytic precursor cells [50–52]. Conditioned media was collected from MG63-A4 and MG63-EV cells. Proliferation of monocytes (Fig. 5a) and formation of TRAP-positive osteoclasts (Fig. 5b) was increased by exposure to 10% conditioned media from ANGPTL4 over-expressing cells. MG63-A4 conditioned medium also increased bone resorption of mature osteoclasts 2.2-fold (Fig. 5c). No individual isoform of ANGPTL4 could reproduce the effect of MG63-A4 conditioned medium on monocyte proliferation or osteoclast-mediated bone resorption (Additional file 2: Figure S2). However, nANGPTL4 did increase the formation of osteoclasts from CD14+ monocytes, causing a 1.89-fold increase in the number of TRAP-positive osteoclasts formed (Fig. 5d, e).

## Discussion

Here we present the first description of expression of ANGPTL4 in human osteosarcoma tissue. Although ANGPTL4 is over-expressed in a range of epithelial tumours [28–35] there is only one other report in primary bone tumours, where it was recently shown that ANGPTL4 is over-expressed in Giant Cell Tumour of Bone [53]. Although we do not have patient or clinical data with which to investigate any correlation of clinical characteristics with ANGPTL4 expression level by immunohistochemistry, we were able to examine this correlation using a publicly available gene expression database. Although there was no correlation between ANGPTL4 expression levels in non-metastatic osteosarcoma versus osteosarcoma that had metastasised, we did find an increase in ANGPTL4 expression in stage 2 disease (osteosarcoma that has spread beyond the bone) versus either stage 1 (osteosarcoma that has not grown outside the bone) or stage 3 (osteosarcoma that has metastasised to another body site). This suggests that ANGPTL4 over-expression may promote primary osteosarcoma infiltration and extension outside the bone.

The magnitude of hypoxic induction of ANGPTL4 varied from 3.4- to 35-fold between the different osteosarcoma cell lines. This variability is a common characteristic of hypoxic secreted factors, as levels of induction will be determined by differences in both cell genotype and expression levels of regulatory enzymes, and is also evident for ANGPTL4 in gastric cancer [54] and uveal melanoma [55] cell lines. We have previously shown that hypoxia-induced gene transcription is regulated by both HIF-1α and HIF-2α in osteosarcoma cells [9]. Reports in other cell lines described ANGPTL4 as regulated solely by HIF-1α [16–18], however hypoxic induction of ANGPTL4 was also regulated by both HIF-1α and HIF-2α in osteosarcoma cells. As hypoxia and HIF correlate with the progression and recurrence of osteosarcoma and are predictive of poor survival [2–8], increased expression of ANGPTL4 in stage 2 pre-metastatic disease is potentially a mediator of this phenotype.

When considering the effect of either ANGPTL4 over-expression or ANGPTL4 knock-down in tumours as diverse as hepatocellular carcinoma [17], gastric cancer [56], colorectal cancer [57, 58], squamous cell carcinoma [59], breast cancer [18] and giant cell tumour of bone [53], the presence of ANGPTL4 is described to promote the tumourigenic phenotype. In agreement with this literature, MG63 cells over-expressing ANGPTL4 exhibited increased proliferation and migration capacity in comparison with control cells, supporting a stimulatory effect of ANGPTL4 on disease progression in osteosarcoma.

The functions of the three different forms of ANGPTL4 are not clearly defined. Although nANGPTL4 is responsible for the regulation of lipid metabolism (20, 22), the biological functions of flANGPTL4, cANGPTL4 and nANGPTL4 with respect to cancer growth, angiogenesis and metastasis are contradictory [60, 61]. As we previously reported for the Soas2 osteosarcoma cell line [16] flANGPTL4 increased the rate of proliferation of MG-63 cells, while nANGPTL4 induced cell migration. However, the amplitude of this response did not match that seen in the ANGPTL4 transfectants. As far as we are aware, this is the first report of any effect of nANGPTL4 in tumourigenesis assays [60].

Similarly, the stimulatory effect of the conditioned media from ANGPTL4 transfectants on osteoclast differentiation and activity was much greater than that observed with any individual isoform. ANGPTL4 has a general stimulatory effect on osteoclast formation and activity. We originally showed that flANGPTL4 increases the amount of bone resorption performed by mature human osteoclasts, without exerting any effect on osteoclast differentiation [16]. We now report that nANGPTL4 promotes the differentiation of human monocytes into TRAP-positive multi-nucleated osteoclasts. Li et al. also recently reported a stimulatory effect of exogenous ANGPTL4 on osteoclast formation from murine bone marrow macrophages or RAW264.7 cells [53]. Similarly, the stimulatory effect of conditioned media collected from Giant Cell Tumour of Bone stromal cells on osteoclast formation was inhibited in ANGPTL4 knock-out cells [53]. In contrast to this positive data, it has been reported that nANGPTL4 inhibits the formation and activity of murine osteoclasts, while the C-terminal and full-length forms had no effect [62]. Although we cannot account for this discrepancy here, it would seem that ANGPTL4 does also correlate with bone erosion in vivo. Rheumatoid arthritis patients with high serum concentrations of ANGPTL4 also have high serum levels of RANKL, a circulating marker of bone erosion [40].

Despite being structurally similar to the angiopoietins, the angiopoietin-like (ANGPTL) family of proteins do not bind either the Tie1 or Tie2 receptor and have no identified cognate receptors, rendering them orphan ligands [60]. Lack of detailed knowledge of the downstream molecular effects of ANGPTL4 and how they are mediated is possibly the major obstruction to furthering our understanding of the role(s) of the individual forms of ANGPTL4 in osteosarcoma and other cancers.

## Conclusions

Our study has shown that over-expression of ANGTPL4 promotes tumourigenic characteristics of osteosarcoma cells including proliferation and migration as well as osteoclast formation and bone resorption activity. These findings highlight ANGPTL4 as a potential molecular target for the treatment of osteosarcoma, inhibition of which could conceivably target the primary tumour, inhibit angiogenesis, reduce metastatic events and prevent bone destruction.

## Additional files

Additional file 1: **Figure S1.** ANGPTL4 knock-down has no phenotypic effect under standard cell culture conditions. (DOCX 162 kb)

Additional file 2: **Figure S2.** Effect of ANGPTL4 on bone resorption performed by mature osteoclasts. (DOCX 94 kb)

## Abbreviations

ANGPTL4: Angiopoietin-like 4
DMOG: Dimethyl oxalyl glycine
HIF: Hypoxia-inducible factor 1-alpha
MCSF: Macrophage colony stimulating factor
RANKL: Receptor activator for nuclear factor kappa B ligand
TMA: Tissue microarray
TRAP: Tartrate-resistant acid phosphatase
