# Supplementary material for: Angiopoietin-like 4 promotes osteosarcoma cell proliferation and migration and stimulates osteoclastogenesis
Source: BMC Cancer. 2018 May 8;18:536. doi: 10.1186/s12885-018-4468-5 (PMC5941625; doi:10.1186/s12885-018-4468-5)
Supplement: Supplementary file 1 — Figure S1. ANGPTL4 knock-down has no phenotypic effect under standard cell culture conditions. (DOCX 162 kb) [file 12885_2018_4468_MOESM1_ESM.docx]

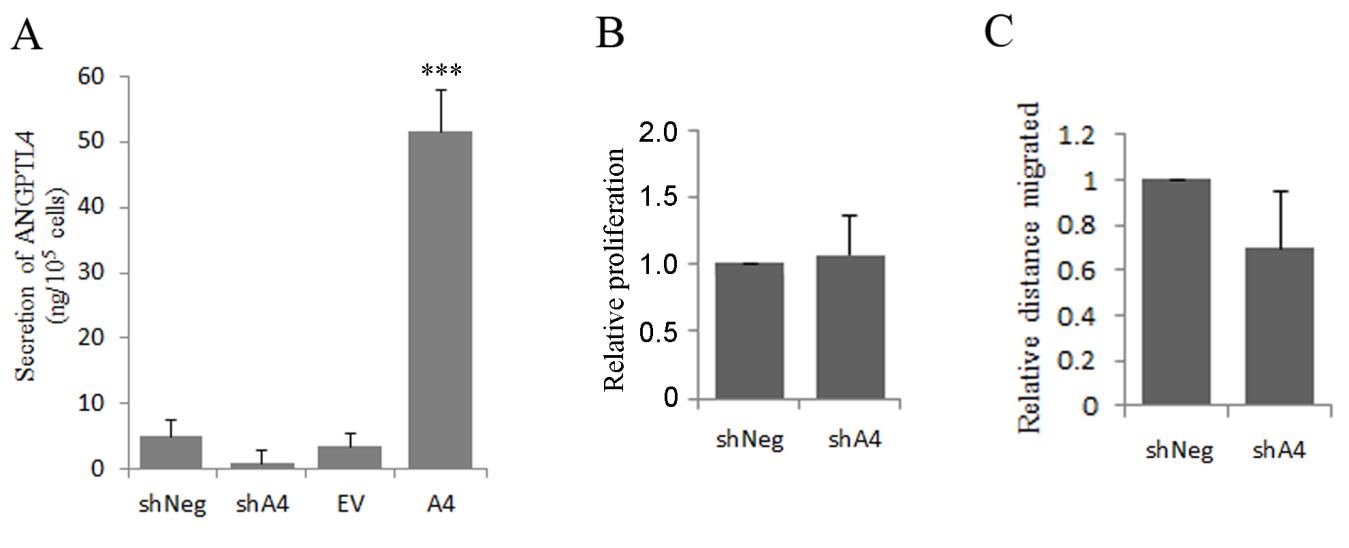


**Figure S1. ANGPTL4 knock-down has no phenotypic effect under standard cell culture conditions.** (a) Quantification of ANGPTL4 secretion by MG-63 cells stably transfected with ANGPTL4 (A4) versus control cells (EV) or with ANGPTL4 shRNA (shA4) versus control cells (shNeg). (b-d) Effect of ANGPTL4 knock-down (shA4) in MG-63 cells with respect to (b) cellular proliferation rate and (c) migration capacity. ***, p<0.001.
