# Supplementary material for: Angiopoietin-like 4 promotes osteosarcoma cell proliferation and migration and stimulates osteoclastogenesis
Source: BMC Cancer. 2018 May 8;18:536. doi: 10.1186/s12885-018-4468-5 (PMC5941625; doi:10.1186/s12885-018-4468-5)
Supplement: Supplementary file 2 — Figure S2. Effect of ANGPTL4 on bone resorption performed by mature osteoclasts. (DOCX 94 kb) [file 12885_2018_4468_MOESM2_ESM.docx]

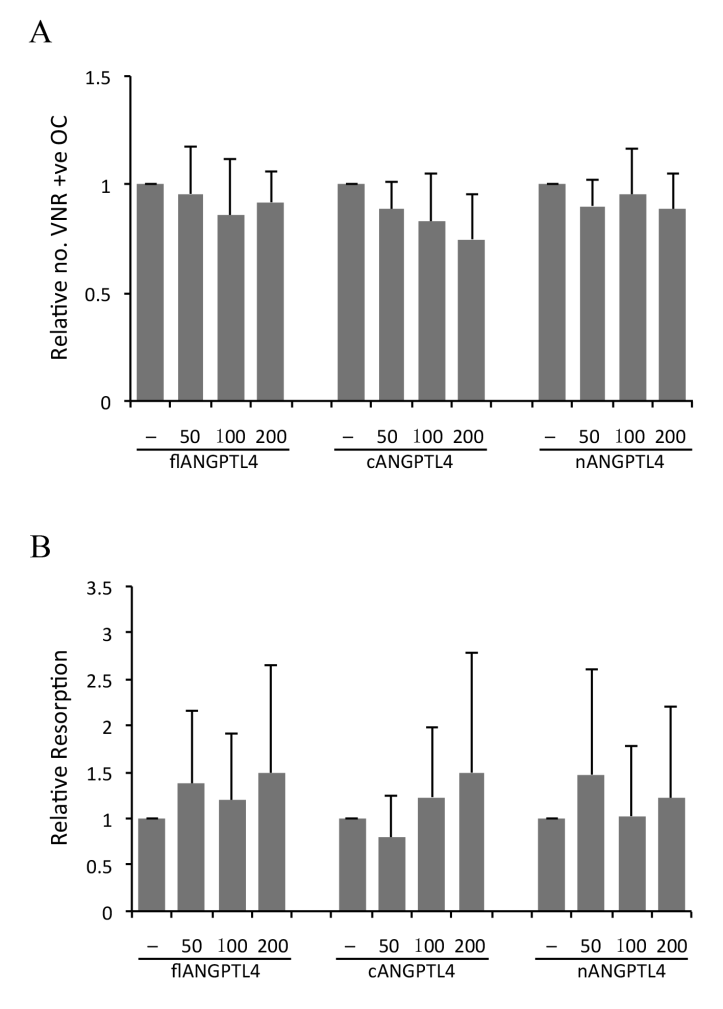


**Figure S2. Effect of ANGPTL4 on bone resorption performed by mature osteoclasts.** (a) Quantification of the number of VNR-positive multi-nucleated osteoclasts present and (b) lacunar resorption per osteoclast following 24 h treatment of mature osteoclasts with different concentrations (50, 100, 200 ng/ml) of flANGPTL4, cANGPTL4 and nANGPTL4.
